# S-type Dissolved Oxygen Distribution along Water Depth in a Canyon-shaped and Algae Blooming Water Source Reservoir: Reasons and Control

**DOI:** 10.3390/ijerph16060987

**Published:** 2019-03-19

**Authors:** Yuwei Huang, Chun Yang, Chengcheng Wen, Gang Wen

**Affiliations:** 1 Faculty of Urban Construction and Environmental Engineering, Chongqing University, Chongqing 400444, China; huang.fade@foxmail.com (Y.H.); c.yang@cqu.edu.cn (C.Y.); 2 Key Laboratory of Northwest Water Resource, Environment and Ecology, MOE, Xi’an University of Architecture and Technology, Xi’an 710055, China; hitchengchengwen@163.com; 3 Shaanxi Key Laboratory of Environmental Engineering, Xi’an University of Architecture and Technology, Xi’an 710055, China

**Keywords:** dissolved oxygen, S-type vertical distribution, metalimnetic oxygen minimum, thermal stratification, algae blooming

## Abstract

Dissolved oxygen (DO) is a crucial indicator of water quality. DO usually shows a monotonic decrease along water depth during thermal stratification in reservoir, whereas metalimnetic oxygen minimum (MOM) is observed in some cases. Although MOM phenomena have been reported in different areas, the characteristics of different reservoirs are greatly different, and few comprehensive studies have been published regarding MOM in Chinese drinking water source reservoirs. The DO distribution along water depth was determined and the detailed reasons were clarified by two-years of field monitoring. In addition the effect of water lifting aerators (WLAs) on DO improvement was investigated in the Lijiahe Reservoir in Northwest China. A typical S-type DO distribution with two anaerobic water layers, below the epilimnion (10–25 m water depth) and above the sediment (bottom water), was observed derived from the decomposition of dead algae or organic matter and the restriction of DO vertical exchange. Moreover, after WLAs’ operation since 10 June 2018, the water body was completely mixed and DO was rich and uniform along water depth by eliminating the water stratification and inhibiting algae growth. The deep understanding of the DO distribution in a deep canyon-shaped reservoir and the technical support for reservoir restoration are meaningful for optimizing reservoir management.

## 1. Introduction

Reservoirs play an important role in supplying water for industry, agriculture, and residential drinking water [1,2]. Thermal stratification occurs at the end of spring or early summer each year, due to the change in water density with temperature at different depths. Generally, the stratified water body is classified into three layers, namely epilimnion, metalimnion and hypolimnion [3]. The formation of metalimnion and its high density gradient has a profound effect on oxycline formation (defined as the depth where the vertical positive gradient of dissolved oxygen in water is greater than 0.2 mg/L/m) and dissolved oxygen (DO) depletion [4,5,6]. DO plays a vital role in biogeochemical cycling and the evolution of ecosystem structure and function [7,8,9]. Low to null DO concentration (anaerobic state) in the middle water layer is harmful to biota (like fish) which require a sufficient oxygen supply. In addition, the low DO concentration in the drinking water reservoir would deteriorate water quality and require a higher cost for water treatment [10]. Anoxic environment formed in the hypolimnion during the thermal stratification period will promote the release of pollutants from the bottom sediments and lead to the recurring deterioration of water quality each year [11,12,13].

However, a more complex situation is observed occasionally. DO profile shows a metalimnetic oxygen minima (MOM) with the characterization of a substantial fraction of DO depleted below the epilimnion in reservoir [14] due to the restriction of mass transfer and vertical DO exchange caused by high density gradients in the metalimnion. Effler et al. have introduced the characteristics and origin of MOM in an eutrophic reservoir in New York, and found that MOM is a recurring phenomenon due to the respiration of relatively high concentrations of phytoplankton biomass within the metalimnion [10]. Wentzyk et al also reported the MOM phenomenon in Rappbode Reservoir located in the Harz Mountains in Northern Germany, and found that the respiration of phycoerythrin-rich *Planktothrix rubescens* was responsible for the oxygen minimum in metalimnion in a low-nutrient drinking water reservoir [15]. Kreling et al further revealed the function of physical transport and oxygen consumption for the development of MOM in a lake, which concluded that more DO was consumed in the metalimnion than that transported to this layer at the same time [16]. The previous studies have preliminarily summarized the formation of MOM mainly due to the following reasons: (1) Higher DO consumption in the metalimnion was mainly attributed to the enhanced decomposition of dead algae or respiration of algae beyond their photosynthetic activity in the metalimnion [15]; (2) the interflow of DO depleted or depleting water also has been claimed to be an important influencing factor for MOM formation [17]; (3) MOM has been related to oxygen depletion at the side wall in some situation, which was advected into the water body of the reservoir on isopycnal water layer [18]; (4) finally, vertical gradient in the water column due to the retardation of diapycnal transfer and vertical exchange led to the reoxygenation difficulty [19]. Although those studies have reported MOM phenomenon in different areas and stated the reasons, the characteristics including the MOM formation mechanisms, the developed thickness and ecological risk of this phenomenon in reservoirs are of great difference with regard to hydrological and climatic conditions [10,15,20,21,22,23]. As far as we known, few comprehensive studies have been published regarding MOM in Chinese deep canyon-shaped drinking water source reservoir, therefore the reason and effect of anoxic area in middle water layer in deep canyon-shaped reservoir are still need to be examined.

In deep stratified reservoirs, the main method of in-situ algae control is artificial mixing, such as pipe mixing and the water-lifting aerators (WLAs). Despite the same efficiency for algae inhibition and water column mixing, pipe mixing fails in energy saving and stratified oxygenation [18]. WLAs are effective in algal cell reduction by 91% and DO concentration improvement above 2 mg/L in hypolimnion [24,25]. In addition, the energy utilization efficiency of the WLAs system was between 5.36% and 7.30% [26]. Those studies have demonstrated that WLAs had pronounced effect on inhibiting algae growth and reducing stratification by mixing and oxygenating function, thus can improve the anoxic environment in hypolimnion. However, the effects and improvements on the anoxia area below epilimnion by WLAs remain unclear.

To shed light on the causes of the anoxia area below epilimnion in Lijiahe Reservoir in Northwest China and the efficiency of WLAs on the DO improvement in this water layer, our investigations were conducted over two years. The objectives of the paper are: (1) to investigate DO distribution along water depth in Lijiahe Reservoir from 2017 to 2018, especially focus on DO depth profile during summer and autumn, to observe the formation of anoxia area below epilimnion; (2) to identify the key processes determining the formation of anoxia area below epilimnion; (3) to investigate the influence of WLAs on DO vertical profile, and confirm the WLAs’ capability to improve the water quality of reservoir.

## 2. Materials and Methods 

### 2.1. Study Site

**Figure 1:**
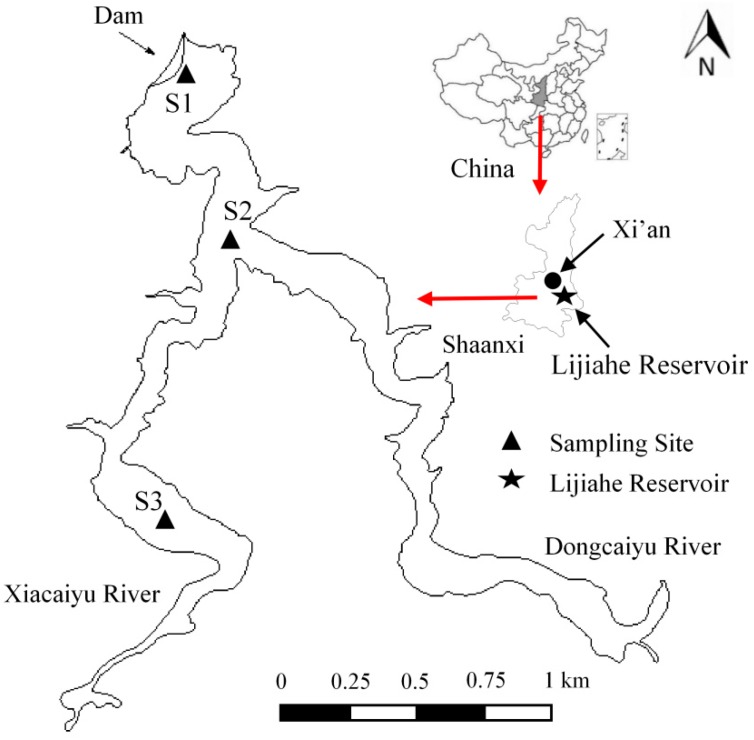
Illustration of Lijiahe Reservoir location in Shaanxi Province, China.

Lijiahe Reservoir (LJHR) (33°59’N, 109°24’E) is a canyon-shaped reservoir located in the southeast of Xi’an city in Shaanxi Province, China (Figure 1), started impounding in 2015. The Xiacaiyu River and Dongcaiyu River are the two main tributaries of the LJHR, with the total catchment area of 362 km^2^ covered by farmland and forest. The reservoir is the raw water source of Xi’an city, with the water area of 2.18 km^2^, the average residence time of 160 days, the mean and maximum depth of 56 m and 80 m, respectively, and a total storage capacity of 5.7 × 10^7^ m^3^. The region has continental monsoon climate with an annual mean precipitation of 720.4 mm and an annual mean temperature of 13.1 °C.

In order to study the effect of side wall on the water quality and DO of LJHR, three representative sampling sites were chosen for water quality monitoring from upstream to the dam of reservoir. S1 is located near the dam with the maximal water depth and in a stagnant state. S2 is located in the transition area between the main area and upstream of reservoir and 1.0 km from the S1 site. S3 is situated in upstream of reservoir and 1.5 km from the S2 site.

### 2.2. Introduction of Water-Lifting Aerators in Lijiahe Reservoir 

**Figure 2:**
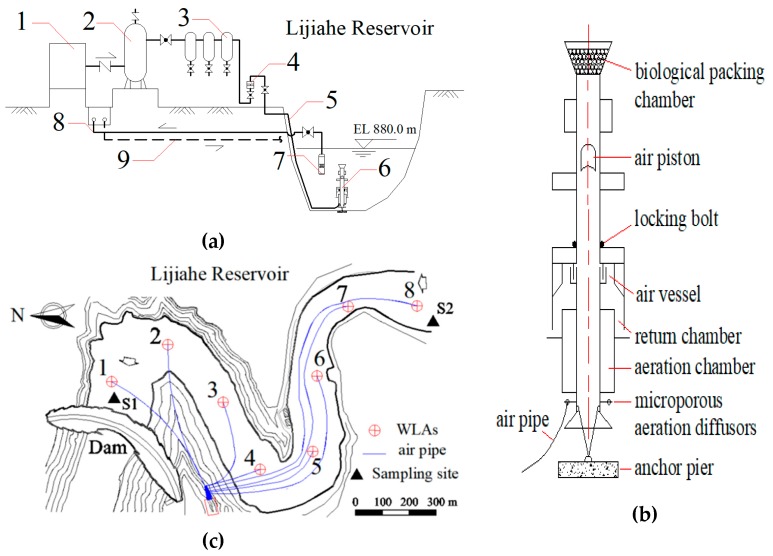
The water lifting aeration system: (**a**) sketch of the water lifting and aeration system, (**b**) layout of the water lifting aerators in Lijiahe Reservoir, (**c**) diagram of the water lifting aerator structure. 1-air compressor; 2-gas holder. 3-air filter; 4-flowmeter; 5-air pipe; 6-WLAs; 7-cooling water pump; 8-cooling pipe; 9-return pipe.

Improved water-lifting aerators (WLAs) were installed in the main area of Lijiahe Reservoir in 2018. The sketch and layout of the WLAs system are shown in Figure 2a,b. The compressed air was delivered to WLAs through gas holder and air filters. The cooling system was designed to control the temperature of the operating system, including cooling water pump, cooling pipe and return pipe. 

The WLAs system consisting of eight water-lifting aerators achieves the coverage of the whole reservoir, whose diagram is provided in Figure 2c. The previous study demonstrated that WLAs system had three distinguished functions: destratification, oxygenation and algae inhibition [24,27,28]. The gas flow of the WLAs system was controlled between 20 and 50 m^3^/h, mainly considering the generation of air piston and the mixing efficiency of water column. The setting of low gas flow was a key step in forming a big air piston and oxygenating the hypolimnion, while high gas flow could inhibit algae growth effectively by account of affecting ecological niche of algae.

The WLAs system was operated from 10th June to 31th September in 2018, covering the period of algae blooming. Sampling point S1 and S2 are situated within the active region of the WLAs system. S3 is located in the inactive region of the WLAs system. The operation of WLAs system achieved the goals of eliminating the oxygen minimum and reducing algae biomass.

### 2.3. Sampling and Analysis

Sampling was performed monthly from January 2017 to December 2018 for two years, and the sampling frequency increased up to weekly during the period of algae blooming. Water samples for algae analyses were collected using the 1 L polyethylene bottle at different depths from the surface (0.5 m below the surface) to the bottom (0.5 m above the sediments). All algae samples were fixed with neutral Lugol’s solution and concentrated after 48 h sedimentation [29]. For algae quantification, enumeration was carried out using an Olympus light microscopy [30,31]. All the experiments were done in triplicate and the presented result was the average value.

Water temperature, DO, turbidity, chlorophyll a (chl-a) and pH were in-situ measured with an increment of every 2–5 m between the surface and the bottom using a Hydro-lab DS5 (HACH, Loveland, CO, USA). All probes of the Hydro-lab DS5 were calibrated monthly by HACH Corporation (Xi’an City, China). Hydrological data including inflow rate, outflow rate, precipitation and water level were obtained from Lijiahe Reservoir Administration Bureau.

### 2.4. Calculation Method of the RWCS/H Index

The RWCS index, firstly proposed by Becker and Xiao [32,33], was used to evaluate the stability of thermal stratification. Meanwhile, considering the effect of water depth on water column stability, the RWCS/H index was introduced to indicate the stability of thermal stratification more effectively, which was calculated as follows:

(1) $$\mathrm{RWCS}/H=\frac{D_{b}-D_{s}}{\left(D_{4}-D_{5}\right)H}$$

where D_b_ is the bottom water density, D_s_ is the surface water density, and D_4_ and D_5_ are the density of pure water at 4 °C and 5 °C, respectively. H is the total water depth [34].

### 2.5. Data Analysis

All statistical tests were performed using SPSS (version, 19.0, IBM Corporation, Armonk, NY, USA), including regression and correlation analyses for oxygen consumption process, and *p* < 0.05 was accepted as being significant [35]. OriginPro (version, 8.5.1, OriginLab Corporation, Northampton, MA, USA) and Surfer (version, 12.0, Golden Software Corporation, Golden, CO, USA) were used for plotting.

## 3. Results and Discussion

### 3.1. DO Distribution along Water Depth over One Year

**Figure 3:**
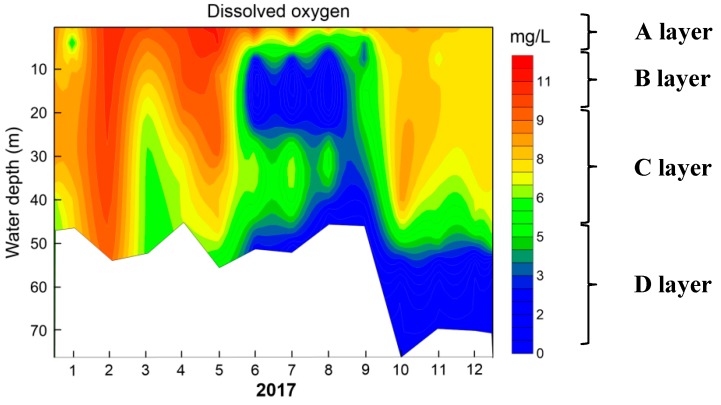
Seasonal DO depth profile of S1 site in Lijiahe Reservoir in 2017.

DO distribution of S1 site along water depth per month during 2017 is shown in Figure 3. There was a seasonal variation in DO concentration all over the year. The water column remained mixed, with the DO concentration kept at about 8.7 mg/L in January and slightly increased up to 9.8 mg/L in February. In spring (March-May), there was only a weak DO difference between the upper and bottom layer. DO concentration decreased slightly from 10 m water depth, but always higher than 6 mg/L. During the period from June to September, vertical DO concentration showed a S-type distribution. The DO concentration reached 11.9 mg/L in the upper 5 m water layer (defined as A layer), then decreased rapidly from 5 m water depth with an average decrease gradient of 2.7 mg/(L·m) in the anoxic layer between 10 and 25 m(defined as B layer). At the lower layer of metalimnion (C layer) DO increased significantly and finally decreased to 0 mg/L near the bottom (D layer). From June to August, DO-depleted water layer (B layer) extended gradually and reached anaerobic state (0 mg/L) in August, with the anaerobic layer thickness of 10 m. In September, the DO concentration increased slightly in the anoxic layer (B layer), with the DO concentration recovered up to 2 mg/L. Table 1 shows that the proportion of anoxia layer increases up to 15.4% in early July and reached the maximum of 62.9% in late August. From October to December, DO concentration showed the same pattern, with the DO concentration maintaining uniform from the water surface to the water depth of 40 m, and then decreased gradually to zero (D layer). For example, in December, the DO concentration remained approximately 8.3 mg/L in the upper 40 m water depth and then rapidly decreased to 0 mg/L at the water depth of 55 m (D layer). DO depth profile of S2 and S3 site in Lijiahe Reservoir shows the same pattern, which can be found in the Supplementary Materials (Figures S1 and S2).

The appearance of anoxic area in D layer is a common phenomenon, which is attributed to the higher DO consumption by the sediments and the small DO vertical transport rate because of the high density gradient in the metalimnion [36]. Generally, DO concentration along the water depth reduces monotonously due to the DO transport limitation by the metalimnion and the enhanced DO demand by the sediments [37,38]. However, in this study, the DO distribution was a kind of S-type distribution with two anoxia water layers, which was remarkable different from previous results [14,16,20,21,22,23,38].

The anoxia area in B layer (between 10 and 25 m water depth) is quite interesting, which appeared from June to September and reached the maximum (in anaerobic state) in the anoxia area in August. The phenomenon is partially similar to the MOM reported in several lakes or reservoirs [16,21]. But it was also slightly different from the MOM, firstly, the anoxia area in B layer can reach anaerobic state (null DO concentration) in August; secondly, the anoxic layer started to form at the position (10 m water depth) above the metalimnion, and extended to the middle of metalimnion (25 m water depth). The detailed reasons for the formation of anoxic area in B layer would be discussed in detail in Section 3.2.

### 3.2. Reasons for the Formation of Anoxic Area in B Layer

#### 3.2.1. Thermal Stratification on the Formation of S-type DO Distribution 

**Figure 4:**
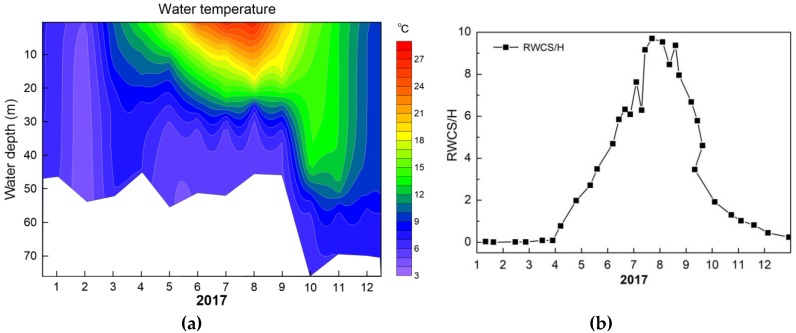
Seasonal water temperature depth profile of S1 site (**a**), thermal stratification index RWCS/H (**b**) in Lijiahe Reservoir in 2017.

The observed temperature profile in water is given in Figure 4a. As shown in Figure 4, during January and February, the temperature profile was uniform along the water depth at around 5 °C. Spring (March–May) was the metalimnion formation period, then stable stratification was formed in August where the highest water temperature reached 29.8 °C at the surface water and decreased down to 5.7 °C above the sediment. The metalimnion was located at water depth from 20 m to 26 m, where water temperature decreased rapidly and remained at approximately 5.7 °C down to the benthic water. From September, the water temperature at surface water was continuously decreased because the air temperature dropped and the water column was uniform. 

**Figure 5:**
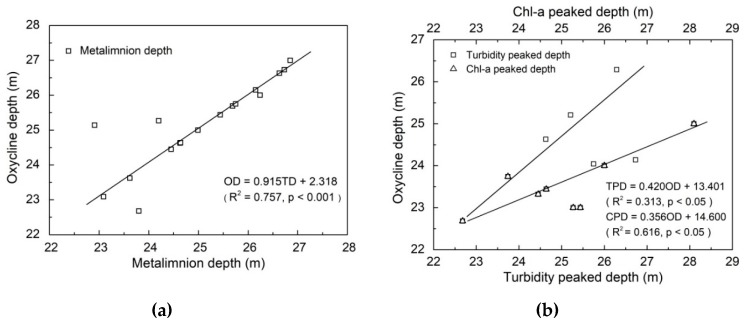
Regression between oxycline depth and metalimnion depth (**a**), chlorophyll a and turbidity peaked depth (**b**) in Lijiahe Reservoir. OD: oxycline depth; TD: metalimnion depth; TPD: turbidity peaked depth; CPD: Chlorophyll a peaked depth.

The thermal stratification index RWCS/H was calculated and presented in Figure 4b. The mixing period was from January to early March, and then thermal stratification began to form during mid-March to May. Thermal stratification index was quite stable from June to September. There was a significant consistency between the metalimnion depth and the oxycline depth (Figure 5a) during the period of metalimnion formation and stabilization (June–August), indicating that there was a positive relationship of metalimnion formation and DO vertical change along water depth. 

Both organic and inorganic matters would accumulate in the equidensity water layer, and then consumed a lot of DO when they were decomposed [39]. In water depth from 20 m to 26 m, turbidity and chlorophyll a were accumulated, whose peak depth was in a good accordance with oxycline depth (Figure 5b). Our results were also similar to those observed in Lake Tahtali [4], where the turbidity increase was closely related to the location of the equidensity water layer. This could be explained by sinking of organic matters, which mainly included dead algae produced in the epilimnion and moved down to the equidensity water layer (the upper layer of metalimnion), where bio-oxidation reduced the DO level below the epilimnion (B layer). Furthermore, the DO gradient diffusion coefficient in water decreased with temperature reduction [12]. In addition, metalimnion could cause high density gradient in the vertical water column due to the remarkable temperature difference, and the formed high density gradient restricted the vertical recirculation of water [4], thus preventing the oxygen recharge. As the result of both the accumulation of organic matters or inorganic matters and the limitation of oxygen recharging ways, the water layer became anoxic. In summary, metalimnion formation resulted in the accumulation of degradable organic carbon or reduced substances and blocked the re-oxygenation of lower water layer and thus led to the formation of anoxia area in B layer. 

#### 3.2.2. The Contribution of Algae Blooming to the Anoxia Area in B Layer

**Figure 6:**
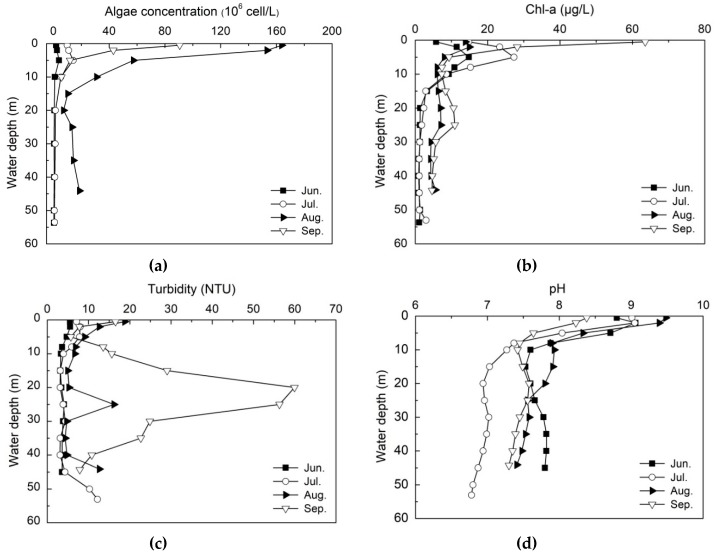
Seasonal algae concentration (**a**), chlorophyll a (**b**), pH (**c**), turbidity (**d**) depth profiles of S1 site in Lijiahe Reservoir from June to September in 2017.

To explore the contribution of algae blooming to the S-type DO distribution, the algae cell concentration, chlorophyll a, turbidity and pH from June to September in 2017 are presented in Figure 6. In July and August 2017, the average algae cell concentration in the surface water layer was 1.43 × 10^7^ cells/L and 1.51 × 10^8^ cells/L, respectively. The peak value reached 2.88 × 10^8^ cells/L on the 22nd of August. During the period of algae blooming, *Cyanophyta* was the dominant algae in the reservoir, accounting for 48% of the total algae, and the dominant algal specie was *Microcystis aeruginosa*; *Chlorophyta* accounted for 31%, and the dominant algal species were *Chlorella*, *Cosmarium* and *Oocystis*; *Bacillariophyta* accounted for about 19%, and *Cyclotella* and *Synedra* were the dominant algal species. The average concentrations of COD_Mn_, total nitrogen (TN) and total phosphorus (TP) in summer were 3.86 ± 0.72 mg/L, 2.42 ± 0.34 mg/L and 0.036 ± 0.037 mg/L, respectively. The higher TN and TP were the main reason responsible for algae blooming [40]. In addition, light intensity was another key factor promoting algae growth, the surface light intensity reached about 42,000 lux, the depth of euphotic zone varied from 2 m to 5 m, and the average depth was 3.55 m.

The vertical concentration of chlorophyll a reduced dramatically below the euphotic zone (3.55 m). Interestingly, chlorophyll a concentration showed an increase about 5–10 μg/L from the water depth 10 m to 25 m. There were two peaks of turbidity in the surface water layer between 0 m and 5 m and in the water depth from 20 m to 25 m, with the first peak value of 18.8 NTU in August and the second peak value of 59.9 NTU in September, respectively. The turbidity in other water layers was remaining at about 3 NTU. pH maintained at approximately 9.35 between 0 m and 3 m, and decreased significantly under the euphotic zone. The pH variation was mainly related to algae photosynthesis, which consumed CO_2_ in water and modified the equilibrium of CO_2_/HCO_3_^−^/CO_3_^2−^ thus caused the water alkaline. The vertical distribution of chlorophyll a concentration was totally consistent with that of algae cell concentration, with the correlation coefficient of 0.94. There was a good consistency between chlorophyll a (Figure 6b), turbidity (Figure 6c) and pH (Figure 6d) in the surface water as well. 

**Figure 7:**
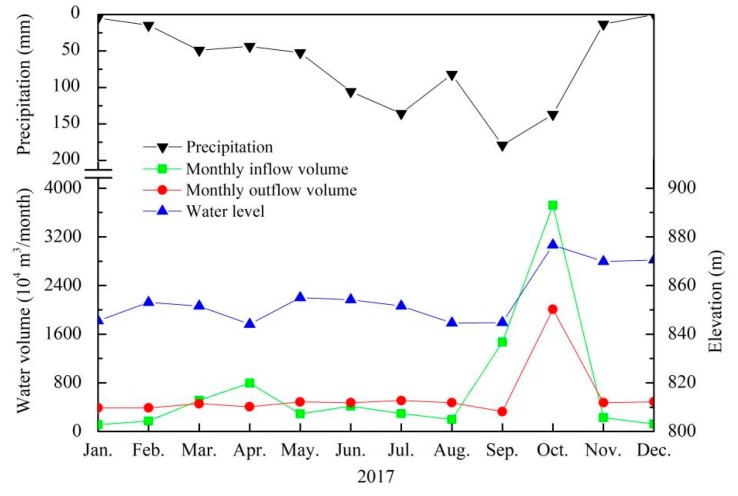
Variations in monthly precipitation, inflow volume, outflow volume, and water level in the Lijiahe Reservoir in 2017.

Due to the strong photosynthesis during algae blooming period (June to August) and small flood inflow to reservoir (Figure 7), the DO in the surface water layer above 2 m depth was supersaturated. Under the condition of sufficient nutrition, the decrease of light intensity below the euphotic zone (3.55 m) limited the photosynthesis and inhibited algae growth, hence the respiration of algae outcompeted their photosynthetic activity, and the decomposition of dead algae and organic matters contributed to DO reduction rapidly from supersaturated to anoxic, resulting in the formation of anoxia area in B layer. 

#### 3.2.3. The Influence of Interflow on the Anoxic Area in B Layer

To clarify the influence of storm runoff on the formation of anoxia area in B layer, variations in monthly precipitation, inflow volume, outflow volume, and water level in Lijiahe Reservoir in 2017 are presented in Figure 7. The total precipitation was 816.98 mm in 2017, with more than 70% of the precipitation occurred between June and October. However, the main inflow occurred in September and October. The outflow volume was relatively stable with the quantity of 4-5 million m^3^ per month for drinking water supply, except that the output volume of 20 million m^3^ in October for flood control. The water level generally maintained at the range of EL 844.06 m to EL 855.04 m, but increased up to EL 876.62 m in October.

The Lijiahe Reservoir was built in Qinglin Mountain, consisting primarily of hills covered with forest with few contaminants, and the water quality was quite good, which was quite similar with that of Heihe Reservoir. Ma and colleagues [28,36] have reported lower storm runoff in Heihe Reservoir could increase the DO in the bottom water. However, higher inflow volumes could also reduce the DO level in the bottom water due to the interflow carrying a lot of suspended solids and organic matters, which increased the oxygen consumption rate in the hypolimnion. Nix et al [17] also pointed out interflow with a high concentration of degradable organic carbons or reduced substances resulted in the formation of MOM in reservoirs. As seen from Figure 7, the inflow volume and outflow volume kept a stable level during 2017 except that in September and October. However, the anoxia area in the metalimnion was firstly formed since June and maintained until September, with the heavy anoxia situation occurring in August. In contrast, in September and October, the anoxia area in B layer was weakened whereas the participation and inflow volume were the highest. Based on the results, it could be concluded that the contribution of interflow to the formation of the anoxic area in B layer was negligible, but probably explained its disappearance.

#### 3.2.4. Side Wall Effect 

**Figure 8:**
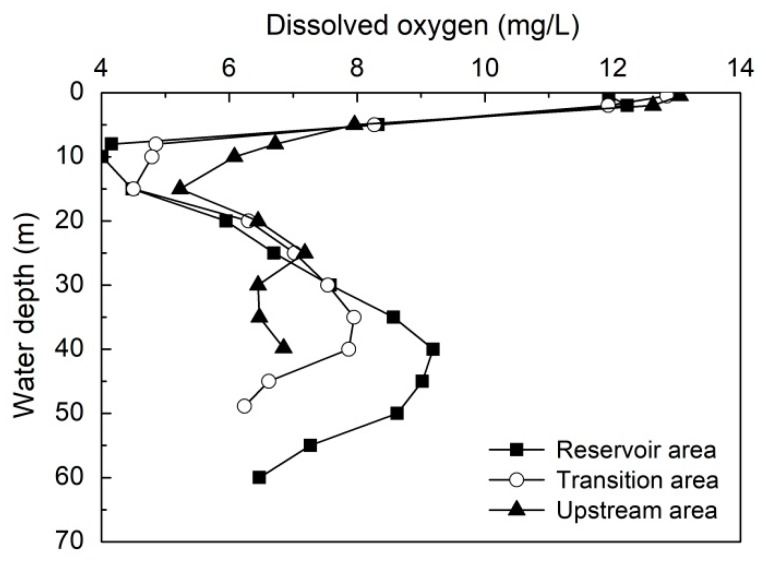
The vertical distribution of DO concentration in the Reservoir area (S1 site), transition area (S2 site) and upstream area (S3 site) in May 2018.

The morphometry of a reservoir may accelerate the DO consumption. For example, Shaprio et al. [41] have found that MOM in a Washington lake was due to the sediment oxygen consumption at the gently slope lake bottom. In general, if the slope of the metalimnetic shore is quite gentle, and the oxygen depletion is enhanced due to the higher ratio of sediment surface area to water volume [16]. For Lijiahe Reservoir, it is a canyon-shaped water body with the slope quite steep. Therefore, the ratio of sediment surface area to water volume is quite low. In addition, Lijiahe Reservoir is located at Qinglin Mountain, and the main component of the wall in B layer consisted of stones. Sediment oxygen uptake should not be the main reason for DO depletion. To confirm this speculation, the vertical distribution of DO concentration in the reservoir area (S1 site), transition area (S2 site) and upstream area (S3 site) in May 2018 were compared and the results are presented in Figure 8. As shown in Figure 8, there were the same pattern in S1, S2 and S3, the vertical DO distribution was a kind of S-type distribution and the anoxia area in B layer ranged from 10 m to 25 m water depth. Comparatively speaking, the anoxia intensity in S3 was weaker although the sampling point is adjacent to the shore. Based on the above results, it demonstrated that there was no influence of side wall on the formation of anoxia area in B layer. 

### 3.3. Improvement of the Anoxic Area by Water Lifting Aerator

**Figure 9:**
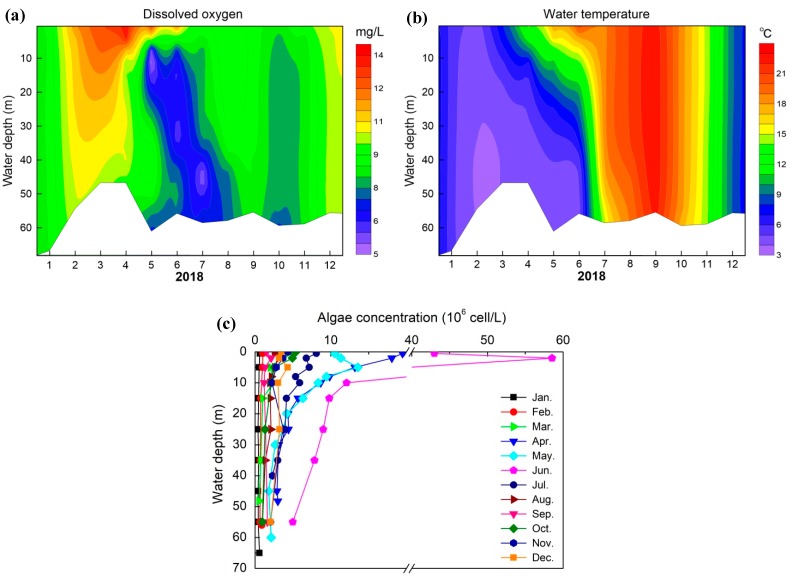
Seasonal DO (**a**), water temperature (**b**), algae concentration (**c**) depth profile of S1 site in Lijiahe Reservoir in 2018.

Mixing oxygenation technology is an effective in situ measure to restore water quality [24,25,42]. In Lijiahe Reservoir, eight WLAs have been installed to improve the water quality. WLAs started running since 10th June, 2018 and stopped at 31th September, 2018. Compared with the same period in 2017, the anoxic and anaerobic phenomena were obviously improved. As shown in Figure 9a, DO depth profile of S1 site showed S-type variation in April, 2018, with obvious decrease of DO at metalimnion at 10 m. DO concentration kept reducing and dropped to minimum at 4.5 mg/L in May at 10 m water depth. After WLAs operation from 10 June, DO recovered rapidly and became vertical uniform distribution, with the DO level above 8.0 mg/L. During the WLAs operation period, the MOM phenomenon in S2 site was eliminated. However, in S3 site the MOM phenomenon could be observed as well in July, 2018 because the S3 site was located in the inactive region of the WLAs system (Figures S3 and S4).

As shown in Figure 9b, thermal stratification occurred in April, 2018, and the metalimnion was formed at 10 m water depth. After WLAs operation, water temperature stratification moved downward and disappeared gradually, then water body was completely mixed (water temperature difference between the surface water and bottom water was less than 0.5 °C) after WLAs’ operation for three months. WLAs took advantage of the air piston formed in ascending tube to push up the low-temperature water in bottom to the surface layer. Water temperature gradient was reduced and correspondingly the restriction of vertical recirculation was weakened, therefore the mass transfer of DO and water mixing were increased. Besides, with WLAs oxygenating the bottom water layer, the DO concentration in bottom water of reservoir increased from 7.5 mg/L to 8.0 mg/L after WLAs operation within one month, which can effectively prevented the continuous reduction of DO concentration in D layer. In addition, the concentration of algae (Figure 9c) reached the peak of 5.9 × 10^7^ cell/L in June in 2 m water depth, but they were reduced significantly after WLAs operation. The decrease of algae concentration in the upper layer reduced the oxygen demand above the metalimnion, in addition, without the restriction of vertical recirculation, the DO concentration in B layer could be supplemented by upper water and maintained at above 8 mg/L during WLAs operation. The depth profile of Chl-a concentration (Figure 9d) was in accordance with the algal concentration, which was decreased remarkably after WLAs operation. 

At present, WLAs have been applied to the water quality improvement in Jin-Pen Reservoir [12,18,26,28,38,43], Shibianyu Reservoir [24], Zhoucun Reservoir [42] and other reservoirs. By destroying the stratification, inhibiting algae growth and restricting the release of nutrients from sediments, the water quality has been effectively improved. In this study, it was firstly reported that WLAs also could improve the DO level in the anoxia area in the water layer close to metalimnion as well.

## 4. Conclusions

(1) A typical S-type DO distribution along water depth was observed from June to September in 2017 in the Lijiahe Reservoir, characterized by two obvious anaerobic water layers below the epilimnion (B layer, 10–25 m water depth) and above the sediment (D layer, bottom water).

(2) Higher oxygen consumption in B layer was the result of the decomposition of dead algae or organic matters and the respiration of algae. Meanwhile, because of the restriction of vertical exchange and diapycnal transfer by metalimnion, anoxia situation below the epilimnion (B layer) was manifested.

(3) The WLAs system improved the anoxic area significantly both below the epilimnion (B layer) and above the sediment (D layer) by eliminating the water stratification and inhibiting algae growth. As a result, water body was completely mixed with uniform oxygen distribution after WLAs operation for three months.

## Figures and Tables

**Table 1:** Anoxia thickness in B layer and proportion in site S1 in Lijiahe Reservoir from June to September in 2017.

Month	Total Depth (m)	Anoxia Layer Thickness (m)	Anoxia Layer Proportion (%)
June	53.7	0	0
July	51.1	15.7	30.7
August	44.1	19.3	43.8
September	44.3	3.2	7.2

## Supplementary Materials

The following are available online at https://www.mdpi.com/1660-4601/16/6/987/s1. Figure S1: Seasonal DO depth profile of S2 site in Lijiahe Reservoir from April to December in 2017; Figure S2: Seasonal DO depth profile of S3 site in Lijiahe Reservoir from April to September in 2017; Figure S3: Seasonal DO depth profile of S2 site in Lijiahe Reservoir in 2018; Figure S4: Seasonal DO depth profile of S3 site in Lijiahe Reservoir from April to September in 2018.
