# Supplementary material for: S-type Dissolved Oxygen Distribution along Water Depth in a Canyon-shaped and Algae Blooming Water Source Reservoir: Reasons and Control
Source: Int J Environ Res Public Health. 2019 Mar 19;16(6):987. doi: 10.3390/ijerph16060987 (PMC6466274; doi:10.3390/ijerph16060987)
Supplement: Supplementary file 1 [file ijerph-16-00987-s001.pdf]

Supplementary Materials

S-type Dissolved Oxygen Distribution Along Water Depth in a Canyon-shaped and Algae Blooming Water Source Reservoir: Reasons and Control

Yuwei Huang [1], Chun Yang [1], Chengcheng Wen [2,3] and Gang Wen [2,3,*]

[1] Faculty of Urban Construction and Environmental Engineering, Chongqing University, Chongqing, 400444, PR China; huang.fade@foxmail.com (Y.H.); c.yang@cqu.edu.cn (C.Y.)
[2] Key Laboratory of Northwest Water Resource, Environment and Ecology, MOE, Xi'an University of Architecture and Technology, Xi'an, 710055, PR China; hitchengchengwen@163.com
[3] Shaanxi Key Laboratory of Environmental Engineering, Xi'an University of Architecture and Technology, Xi'an, 710055, PR China
* Correspondence: hitwengang@163.com Tel.: +86-29-82207886; Fax: +86-29-82202729

List of Contents

Figure S1. Seasonal DO depth profile of S2 site in Lijiahe Reservoir from April to December in 2017

Figure S2. Seasonal DO depth profile of S3 site in Lijiahe Reservoir from April to September in 2017

Figure S3. Seasonal DO depth profile of S2 site in Lijiahe Reservoir in 2018

Figure S4. Seasonal DO depth profile of S3 site in Lijiahe Reservoir from April to September in 2018

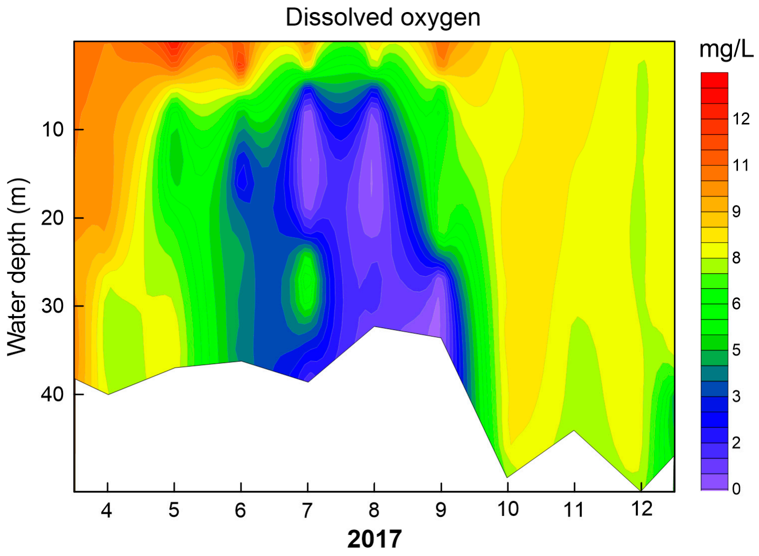

Figure S1. Seasonal DO depth profile of S2 site in Lijiahe Reservoir from April to December in 2017.

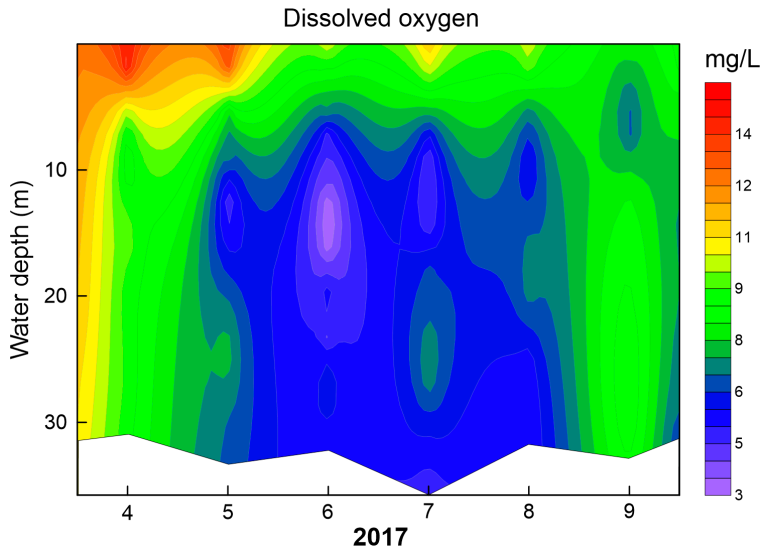

Figure S2. Seasonal DO depth profile of S3 site in Lijiahe Reservoir from April to September in 2017.

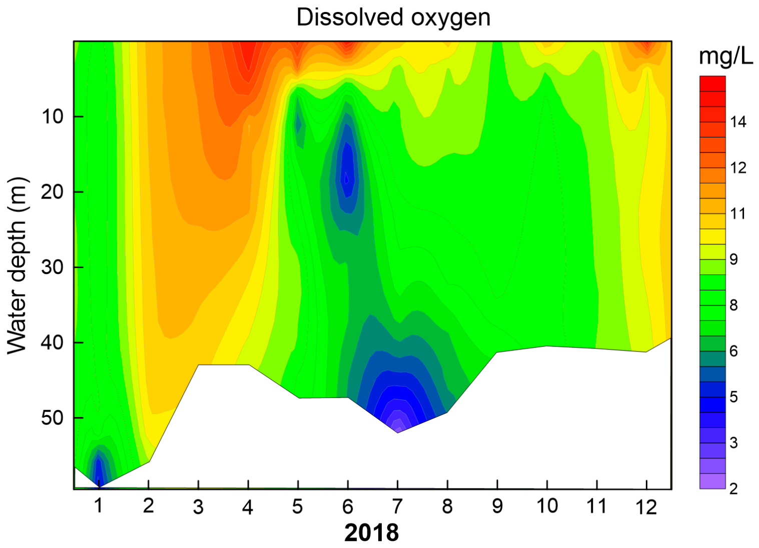

Figure S3. Seasonal DO depth profile of S2 site in Lijiahe Reservoir in 2018.

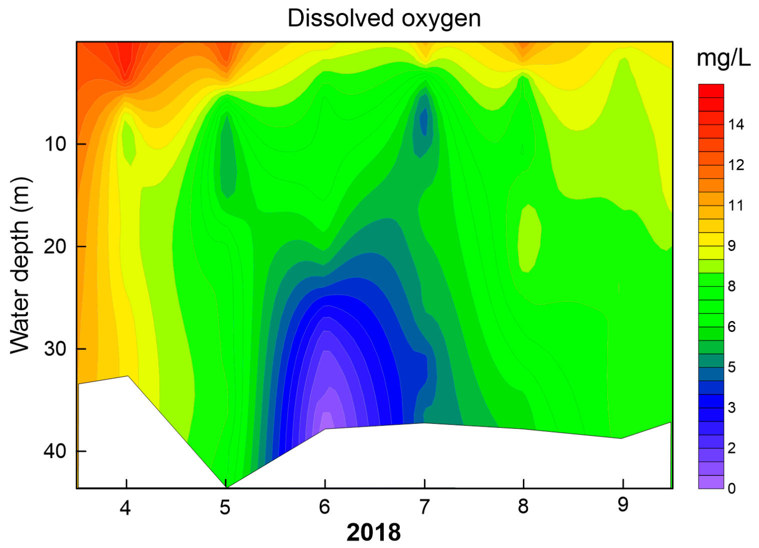

Figure S4. Seasonal DO depth profile of S3 site in Lijiahe Reservoir from April to September in 2018.
